# Surgery as a Viable Alternative First-Line Treatment for Prolactinoma Patients. A Systematic Review and Meta-Analysis

**DOI:** 10.1210/clinem/dgz144

**Published:** 2019-10-29

**Authors:** Amir H Zamanipoor Najafabadi, Ingrid M Zandbergen, Friso de Vries, Leonie H A Broersen, M Elske van den Akker-van Marle, Alberto M Pereira, Wilco C Peul, Olaf M Dekkers, Wouter R van Furth, Nienke R Biermasz

**Affiliations:** 1 Department of Neurosurgery, Leiden University Medical Center, University Neurosurgical Center Holland, ZA Leiden, The Netherlands; 2 Department of Clinical Epidemiology, Leiden University Medical Center, ZA Leiden, the Netherlands; 3 Department of Medicine, Division of Endocrinology, Leiden University Medical Center, ZA Leiden, the Netherlands; 4 Department of Medicine, Center for Endocrine Tumours Leiden, Leiden University Medical Center, ZA Leiden, the Netherlands; 5 Department of Endocrinology, Diabetes and Nutrition, Charité Universitätsmedizin Berlin, corporate member of Freie Universität Berlin, Humboldt-Universität zu Berlin, and Berlin Institute of Health, Berlin, Germany; 6 Department of Biomedical Data Sciences, Section Medical Decision Making, Leiden University Medical Center, ZA Leiden, the Netherlands; 7 Department of Neurosurgery, Haaglanden Medical Center, University Neurosurgical Center Holland, VA The Hague, The Netherlands

**Keywords:** Dopamine agonist, Prolactinoma, Surgery

## Abstract

**Context:**

The improved remission and complication rates of current transsphenoidal surgery warrant reappraisal of the position of surgery as a viable alternative to dopamine agonists in the treatment algorithm of prolactinomas.

**Objective:**

To compare clinical outcomes after dopamine agonist withdrawal and transsphenoidal surgery in prolactinoma patients.

**Methods:**

Eight databases were searched up to July 13, 2018. Primary outcome was disease remission after drug withdrawal or surgery. Secondary outcomes were biochemical control and side effects during dopamine agonist treatment and postoperative complications. Fixed- or random-effects meta-analysis was performed to estimate pooled proportions. Robustness of results was assessed by sensitivity analyses.

**Results:**

A total of 1469 articles were screened: 55 (10 low risk of bias) on medical treatment (n = 3564 patients) and 25 (12 low risk of bias) on transsphenoidal surgery (n = 1836 patients). Long-term disease remission after dopamine agonist withdrawal was 34% (95% confidence interval [CI], 26-46) and 67% (95% CI, 60-74) after surgery. Subgroup analysis of microprolactinomas showed 36% (95% CI, 21-52) disease remission after dopamine agonist withdrawal, and 83% (95% CI, 76-90) after surgery. Biochemical control was achieved in 81% (95% CI, 75-87) of patients during dopamine agonists with side effects in 26% (95% CI, 13-41). Transsphenoidal surgery resulted in 0% mortality, 2% (95% CI, 0-5) permanent diabetes insipidus, and 3% (95% CI, 2-5) cerebrospinal fluid leakage. Multiple sensitivity analyses yielded similar results.

**Conclusions:**

In the majority of prolactinoma patients, disease remission can be achieved through surgery, with low risks of long-term surgical complications, and disease remission is less often achieved with dopamine agonists.

Prolactinomas compose around 50% (95% confidence interval [CI], 32-66) of all pituitary adenomas and are characterized by an overproduction of prolactin, resulting in hypogonadism with symptoms such as galactorrhea, subfertility, loss of libido, and osteoporosis, and general symptoms such as metabolic and psychological problems and fatigue (1). The majority of prolactinomas are microadenomas (≤10 mm; 95% CI, 63-85) and tend to occur more often in women than in men with a peak incidence between 20 and 50 years of age (1–6). The goal of treatment is to relieve symptoms, normalize prolactin levels, induce tumor shrinkage and restore gonadal status, which can be achieved through medical therapy with dopamine agonists or surgical resection or a combination of both (7).

Before the introduction of dopamine agonists for prolactinomas, microscopic surgical resection of pituitary adenomas was the mainstay of treatment (8). The introduction of bromocriptine in the early 1970s resulted in a shift toward less invasive medical treatment (9). Cabergoline was introduced in the 1990s, which, compared with bromocriptine, resulted in better control of prolactin levels, fewer side effects, and improved health-related quality of life (HRQoL) (10, 11). Meanwhile, surgical techniques improved and in 1992 the first series of endoscopically operated pituitary adenoma patients was described (12). The current endoscope is able to provide the surgical team with a panoramic view and instruments have become more delicate than 2 decades ago. Therefore, surgical outcomes of current endoscopic surgery may be similar and possibly superior with less complications, at least in centers of excellence (13, 14). Current guidelines for prolactinoma describe dopamine agonist treatment as primary treatment and (endoscopic) transsphenoidal surgery as a secondary alternative in resistant or intolerant patients (7). Although treatment with dopamine agonists results in normalized prolactin levels and relief of symptoms in the vast majority of patients, withdrawal of dopamine agonists is only possible in a selection. Thus, patients should anticipate long-term medical therapy (15). A wide range of mild side effects has been reported, of which impulse control disorders are rare but potentially devastating adverse events (10, 16). Depending on tumor size and surgical experience, the disease remission and complication rates of transsphenoidal surgery may be deducted from surgical series of pituitary adenomas other than prolactinoma (17, 18). Especially clearly visible microadenomas and small, noninvasive macroadenomas may have a high likelihood for successful resection with low complication rates (17).

The improved disease remission and decreased complication rates of current transsphenoidal surgery warrants reappraisal of the position of surgery as a viable alternative to dopamine agonists in the treatment algorithm of prolactinomas. Therefore, we performed a systematic review and meta-analysis of observational and randomized studies to evaluate long-term disease remission rates after dopamine agonist withdrawal and transsphenoidal surgery. In addition, we assessed biochemical control and side effects during dopamine agonist treatment, complications after transsphenoidal surgery, and HRQoL and cost-effectiveness for both treatment modalities.

## Methods

### Search strategy and study selection

This systematic review and meta-analysis was reported according to the Preferred Reporting Items for Systematic Reviews and Meta-Analyses statement (19).

#### Data Sources and Searches

Publications were retrieved from the following databases on April 13, 2017: PubMed, Embase, Web of Science, Cochrane, Emcare, PsycINFO, Academic Search Premier, and Central. Search terms were formulated related to: 1) the patient population: “prolactinoma”; 2) the intervention: “transsphenoidal surgery” and/or “dopamine agonists”; and 3) outcomes: “control,” “remission,” “HRQoL,” “costs,” “side effects;” and “complications.” Moreover, search terms were used to exclude articles with only animals, case reports, reviews, and studies in other languages than English, Dutch, German, or French. The literature search was updated on July 13, 2018. A more detailed description of the search strategy for PubMed and the other databases is shown in Reference (20). Reference lists of included articles were scanned for additional relevant studies. Full-text articles of potentially relevant studies not available through the university library were requested from the authors.

#### Study selection

**Figure 1.**
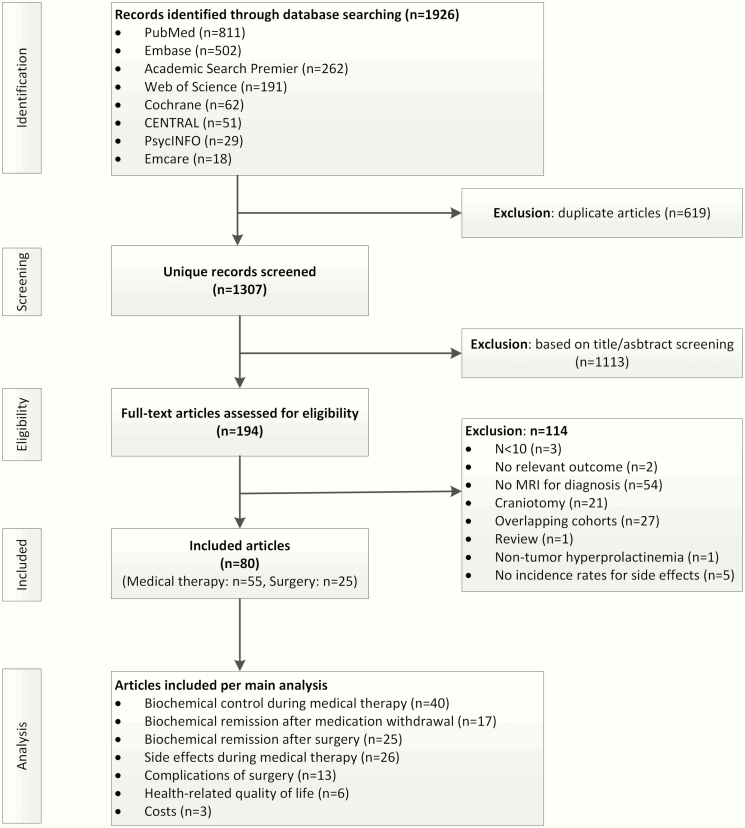
Screening and inclusion of articles. Abbreviation: MRI, magnetic resonance imaging.

Articles were included after 2 rounds of screening by 2 researchers independently (A.H.Z.N. and L.H.A.B.; I.M.Z. and F.d.V.): round 1) title and abstract and round 2) full-text. In case of disagreement, a third reviewer (N.R.B.) was consulted and the disagreement was solved based on consensus. Inclusion criteria were the following: randomized and observational studies of prolactinoma patients undergoing transsphenoidal surgery or receiving treatment with dopamine agonists and describing at least 1 of the following outcomes: disease remission after dopamine agonist withdrawal or transsphenoidal surgery, biochemical control or side effect during dopamine agonist treatment, complications after transsphenoidal surgery, HRQoL, or outcomes of economic analysis. Both comparative as well as single-arm studies were considered. Studies with fewer than 10 patients were not considered; neither were studies diagnosing prolactinoma without magnetic resonance imaging proof of an adenoma, with intramuscular dopamine agonists or radiotherapy as primary treatment, with prolactin levels assessed by dynamic function tests, and with subjects younger than 16 years or pregnant. If multiple studies described the same cohort, only the largest cohort was included. Fig. 1 describes the flowchart of article screening and inclusion.

### Data extraction and quality assessment

Data extraction from the included articles was performed by two independent reviewers (A.H.Z.N. and L.H.A.B.; I.M.Z. and F.d.V.). If available, information was extracted on study design, study period, in- and exclusion criteria (including indication for treatment), and population characteristics: demographics, percentages micro- and macroprolactinomas, previous treatments, prolactin levels at diagnosis, symptoms at diagnosis, and pituitary deficits at diagnosis. In addition, information was extracted on the treatment: surgical technique, type of dopamine agonist, treatment scheme and duration, and indication for dopamine agonist withdrawal. The outcomes of interest were extracted, including mode and moment of measurement. Outcomes of interest for the meta-analysis were: biochemical control and systematically assessed side effects (ie, not anecdotal) during dopamine agonist therapy, long-term (defined as ≥1 year) disease remission after withdrawal of dopamine agonist therapy, short-term (defined as <1 year) and long-term disease remission after transsphenoidal surgery, and postoperative complications. Where possible, data extraction on outcomes was performed separately for patients with micro- (adenoma <1 cm) and macroprolactinomas (adenoma ≥1 cm) year and for giant prolactinomas (adenoma ≥4 cm). Outcomes of interest for the systematic review were HRQoL and cost-effectiveness. More detailed information on the data extraction scheme is provided in Reference (20).

#### Risk of bias assessment

The following aspects were assessed: 1) consecutive patient inclusion, 2) description of surgical and/or medical treatment, 3) description of indication for surgery or dopamine agonist withdrawal), and 4) loss to follow-up <10%. Because none of the included studies directly compared surgery with medical therapy, this was not included in the risk of bias assessment. Subgroup analysis was performed with studies scoring a low risk of bias on at least three out of four points for the primary outcome. The risk of bias assessment was modified for studies on HRQoL. A more detailed description of the criteria for the risk of bias assessment is given in Reference (20).

#### Data analysis and presentation

Results were pooled using fixed-effects (<5 studies included per specific outcome, in which case the between study variance cannot be estimated reliably) or random-effects (≥5 studies) meta-analysis. Random-effects estimates were calculated using the DerSimonian and Laird method (21). Pooled results are reported as percentages with 95% CI. Pooled percentages were estimated for the primary outcome biochemical disease remission and reported separately for studies describing results after dopamine agonist withdrawal and surgery. In addition, pooled percentages were estimated for the following outcomes: biochemical control and side effects during medical treatment, short-term disease remission after transsphenoidal surgery, and complications of surgery (22). A Freeman-Tucky double arcsine transformation was performed to include studies with 0% or 100% outcomes (22). If possible, subgroup analysis was performed for patients with a microprolactinoma, macroprolactinoma, and giant prolactinoma. In addition, multiple sensitivity analyses were performed, including only studies on treatment with cabergoline, second withdrawal attempts, endoscopic or microscopic transsphenoidal surgery, clearly reported minimum follow-up of 1 year, or with a low risk of bias. Case control studies were used to compare prolactinoma patients and controls for prevalence of cardiac valve disease, estimating odds ratios. Where needed, variables with different units were converted to the same unit (eg, prolactin levels). *I*^2^ statistics were used for quantification of between-study heterogeneity. Analyses were performed with Stata version 14.1 (Statacorp). Effect measures on HRQoL and cost-effectiveness were not pooled because of the large heterogeneity of the described outcomes. 

### Role of funding sources

This study was supported by The Netherlands Organisation for Health Research and Development program for Efficiency Studies (80-84300-98-82021). The corresponding author had full access to all the data in the study and had final responsibility for the decision to submit for publication.

## Results

### Study selection and study characteristics

A total of 1469 unique articles were retrieved from the electronic databases, of which 169 were read full-text, and 55 were included on dopamine agonist treatment (n = 3564 patients) and 25 on surgery (n = 1836 patients) (Fig. 1). No studies directly comparing medical with surgical treatment were found. Summarized baseline characteristics of the included studies are described in Table 1 and Reference and information on the individual studies in Reference (20). Regarding medical therapy, patients were treated for a median period of 24 (range, 1-162) months and followed after medication withdrawal for a median period of 12 (range, 2-90) months. Regarding surgery, patients were followed for a median period of 22 (range, 3-135) months. Of the included studies, 10 on medical therapy and 12 on surgical treatment were classified as low risk of bias Reference (20). In particular, those lost to follow-up were poorly reported.

**Table 1.** Baseline Characteristics of Included Studies

	Surgery Studies		Dopamine Agonist Studies	
No. of studies	Total (n = 25)	Missing	Total (n = 55)	Missing
Total number of participants	1836	0	3564	0
Median number of participants per study (range)	63 (11–220)	0	36 (10–694)	0
Median % female (range)	69% (0–100)	5	70% (0–100)	4
Median mean age (range), years	34 (25–43)	7	41 (33–48)	7
Median % microadenoma (range)	43% (3–100)	1	33% (0–100)	5
Median mean tumor diameter (range), mm	17.5 (7–35)	8	27.6 (1.8–71.8)	28
Median % invasive tumors (range)	18% (0–64)	3	86% (0–100)	35
Median % apoplexy (range)	9% (2–28)	12	4% (0–17)	46
Median mean baseline prolactin (range), µg/L	363 (23–7022)	17	976 (53–28,465)	11
Median % previous dopamine agonist treatment (range)	59% (0–100)	8	0% (0–100)	18
Median % intolerance (range)	18% (0–46)	11	0% (0–100)	28
Median % resistant (range)	21% (0–88)	11	0% (0–100)	27
Median % recurrence (range)	0% (0–9)	20	0% (0–100)	26
Median % endoscopic surgery (range)	45% (0–100)	6	-	
Median mean treatment length (range), mo	-		24 (1–162)	18
Median long-term follow-up, mo	22.3 (1–93)	12	12 (3–135)	26

### Disease remission after dopamine agonist withdrawal and after transsphenoidal surgery

**Figure 2.**
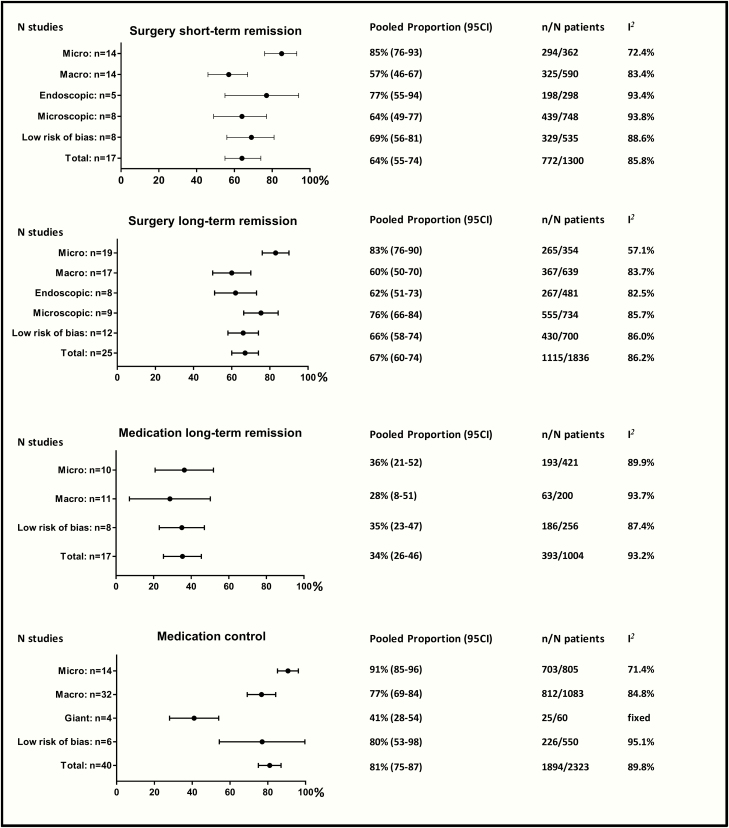
Pooled event rates for dopamine agonists and transsphenoidal surgery.

Long-term disease remission after dopamine agonist withdrawal was achieved in 34% (95% CI, 26-46; 17 studies) of patients, compared with 67% (95% CI, 60-74; 25 studies) of patients after transsphenoidal surgery. Similar results were found restricting to studies with a low risk of bias. In the sensitivity analysis only including studies that defined long-term remission as remission at least after a 1-year follow-up after dopamine agonist withdrawal or surgery, 37% (95% CI, 19-57; 7 studies) of patients achieved long-term remission after medication withdrawal and 74% (95% CI, 62-83; 7 studies) after surgery. In microprolactinoma patients, long-term remission was 36% after medication withdrawal (95% CI, 21-52; 10 studies) and 83% after surgery (95% CI, 76-90; 19 studies). Similar results were found for patients solely treated with cabergoline (32%; 95% CI, 18-48; 8 studies). After a second withdrawal attempt, 31% of patients achieved remission (95% CI, 19-45; 2 studies). In macroprolactinoma, the percentage of long-term remission after dopamine agonist withdrawal was 28% (95% CI, 8-51; 11 studies) and after transsphenoidal surgery 60% (95% CI, 50-70; 17 studies). Remission rates were similar for endoscopically and microscopically operated patients. No studies reported remission rates after dopamine agonist withdrawal or transsphenoidal surgery for giant prolactinoma. Detailed results are depicted in Fig. 2 and Reference (20).

### Control of hyperprolactinemia during medical therapy

Normalization of prolactin levels during treatment with dopamine agonists occurred in 81% (95% CI, 75-87; 40 studies) of patients. Similar results were found restricting to studies with a low risk of bias. In subgroup analyses, these percentages were 91% (95% CI, 85-96; 14 studies) for microprolactinoma patients and 88% (95% CI, 82-94; 24 studies) for patients solely treated with cabergoline. These percentages were lower for macroprolactinoma patients (77%; 95% CI, 69-84; 32 studies) and patients with giant prolactinomas (41%; 95% CI, 28-54; 4 studies). Detailed results are depicted in Fig. 2 and Reference (20).

### Side effects of medical therapy

**Figure 3.**
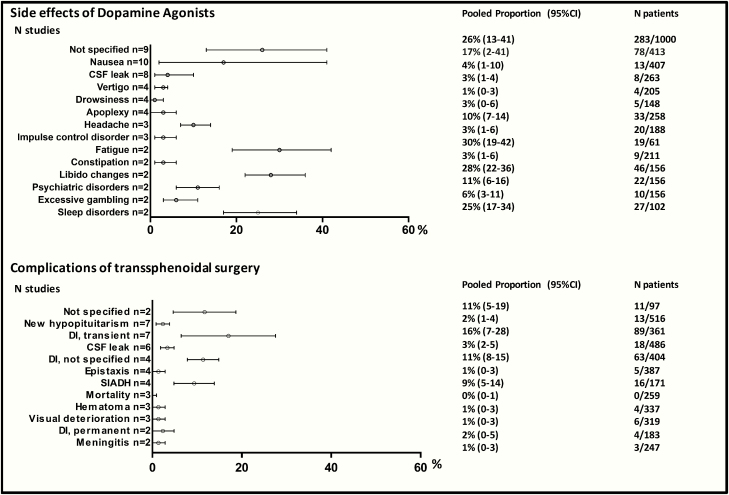
Pooled event rates of side effects of dopamine agonists and complications of transsphenoidal surgery. CSF, cerebrospinal fluid; DI, diabetes insipidus; SIADH, syndrome of inappropriate antidiuretic hormone secretion.

The most frequently occurring side effects as reported by at least 2 studies were: fatigue 30% (95% CI, 19-42), libido changes resulting from side effects 28% (95% CI, 22-36), sleep disorders 25% (95% CI, 17-34), and nausea 17% (95% CI, 2-41). The following severe side effects occurred: impulse control disorders 3% (95% CI, 1-6), excessive gambling 6% (95% CI, 3-11), and cerebrospinal fluid leakage 4% (95% CI, 1-10) Unspecified side effects occurred in 26% (95% CI, 13-41; 9 studies) of patients. Pooled percentages of other reported side effects in the literature are presented in Fig. 3 and Reference (20). Compared with healthy controls, the odds ratio for clinically relevant valve disease was 1.24 (95% CI, 0.59-2.60) and for subclinical valve disease 0.73 (95% CI, 0.53-1.00).

### Complications after surgery

Surgery related mortality occurred in 0% of patients (95% CI, 0-1; 3 studies). The following severe complications occurred: permanent diabetes insipidus 2% (95% CI, 0-5), meningitis 1% (95% CI, 0-3), and cerebrospinal fluid leakage 3% (95% CI, 2-5). The most frequently occurring complications as reported by at least 2 studies were endocrine complications: transient diabetes insipidus 16% (95% CI, 7-28) and syndrome of inappropriate antidiuretic hormone secretion 9% (95% CI, 5-14). Hypopituitarism occurred in 2% of patients only (95% CI, 1-4) and only 4 studies described the involved axis. Patients suffered from hypocortisolism (1%-2%), hypogonadism (3%-6%), and hypothyroidism (1%-6%), whereas information on the other axes was not reported (23–26). Although surgical complications may differ between patients with microprolactinoma and macroprolactinoma, only 2 studies reported results on complications in microprolactinoma: transient diabetes insipidus (n = 2, 18%), new hypopituitarism (n = 1, 4%), and syndrome of inappropriate antidiuretic hormone secretion (n = 1, 4%) (25, 27). Pooled percentages of other reported complications are presented in Fig. 3 and Reference (20).

### HRQoL

HRQoL was measured in only 6 studies and 315 patients (4, 11, 28–31). Risk of bias for included studies was low for 4 studies (20). Compared with controls, patients tend to have lower to similar HRQoL scores before treatment (29). Postoperatively patients reported similar or lower scores for different HRQoL domains compared with controls (28, 32). During medical therapy and after dopamine agonist withdrawal, patients scored consistently lower on all HRQoL scores compared with controls (11, 30, 31). Detailed results are described in Reference (20).

### Economic analysis

Three studies performed economic analysis using decision tree analysis and numbers from the literature on disease control, remission rates, and HRQoL (utility scores) in prolactinoma patients (33–35). Two cost-effectiveness studies found that surgery dominates treatment with bromocriptine and cabergoline, meaning less costly and more effective (33, 34). One study assessed only costs and not cost-effectiveness and found that surgery was more expensive than dopamine agonist treatment (35). Detailed results are described in Reference (20).

## Discussion

Current guidelines prefer dopamine agonist treatment over surgical treatment and only advise surgery in cases of intolerance to dopamine agonists or dopamine agonist-resistant prolactinoma, although outcomes of both treatment modalities have never been systematically assessed. This meta-analysis of available literature shows that surgery in prolactinomas is reaching disease remission in the majority of patients, especially in microprolactinoma patients, with a low risk of long-term complications, including hypopituitarism, and therefore could be revisited as a viable alternative to medical treatment. In addition, recent economic analyses suggest that surgery dominates medical treatment on costs and effectiveness. An important drawback of surgery is the risk of permanent severe complications and mortality, which occur in 3% and 0% of cases, respectively, which is comparable to literature on pituitary surgery in other types of pituitary adenomas (8, 36, 37). Next, this meta-analysis elaborates on the comparison between ongoing dopamine agonist treatment and transsphenoidal surgery. Biochemical control during dopamine agonist treatment (91%) and disease remission after surgery (83%) was somewhat comparable for microadenomas, but in macroadenomas medical treatment was more efficacious (77%) in achieving disease control than surgery (60%). Although the majority of side effects during medical treatment are not harmful (eg, headache, nausea, constipation), these symptoms may still impair a patient’s HRQoL (11, 31). In addition, patients may suffer from more serious adverse events, such as impulse control disorder sand uncontrolled hypersexuality (16, 38). Cardiac valve disease was not related to dopamine agonist at doses used for prolactinoma patients. Although the limited data on HRQoL suggest a preference for surgery, the incomparability between drug side effects and surgical complications limits the strength of evidence for this comparison.

Long-term disease remission was clearly lower after dopamine agonist withdrawal (34%) than after transsphenoidal surgery (64%). In accordance, a previous meta-analysis including studies on hyperprolactinemia with and without an evident prolactinoma reported even lower (21%) long-term remission rates after dopamine agonist withdrawal (15). Confirmation of the fairly low chance to achieve disease remission with medical treatment is a clinically relevant conclusion that will adjust the expectations of patients and doctors, particularly because the current Endocrine Society guideline states that the risk of recurrence after withdrawal ranges from 26% to 69% depending on prolactin levels at diagnosis and tumor size (7).

Current guidelines describe dopamine agonists as first-line treatment for prolactinoma patients (7). Therefore, in most studies, transsphenoidal surgery was reserved for patients with less favorable prognoses, such as intolerance to dopamine agonists, dopamine agonist-resistant tumor, recurrent tumor after dopamine agonist withdrawal, or tumor with less favorable characteristics (eg, invasiveness, apoplexy). Studying more comparable patients, one may assume that surgical remission rates will be even higher and complication rates lower. This is confirmed in our subgroup analysis comparing both treatment modalities in microprolactinoma patients. In addition, these results were confirmed in multiple sensitivity analyses. Recently, independent centers have proposed that surgery may be considered as a viable first treatment choice for microprolactinoma patients (17, 39). Best candidates for transsphenoidal surgery have enclosed microadenomas not located laterally from the gland because tumor with cavernous sinus invasion have an increased chance of venous bleedings (39). Our meta-analysis results indeed show that surgery is not the preferred treatment for patients with an invasive prolactinoma because postoperative remission rates are less favorable. Furthermore, remission rates after endoscopic transsphenoidal surgery might be affected by the different experience levels of groups, knowing that this technique has a steep learning curve (40, 41). Presurgical treatment with dopamine agonists may result in fibrosis of the prolactinoma, resulting in suboptimal surgical outcomes (42).

### Strengths and limitations

A major limitation of this meta-analysis is the lack of randomized trials and observational studies directly comparing dopamine agonist treatment with transsphenoidal surgery. This means that a formal comparison between medical and surgical treatment was cumbersome because by comparing only single-arm studies, confounding cannot be accounted for properly. For example, compared with studies reporting outcomes of patients treated with dopamine agonists, patients treated by transsphenoidal surgery had a lower median prolactin level and a smaller tumor diameter on study level. This might partially be explained by some surgically treated patients being pretreated with dopamine agonists. In addition, there was a great deal of heterogeneity in dopamine agonists used, treatment length, and withdrawal criteria. The number of studies in some analyses is relatively small and therefore the estimated pooled proportion may be less accurate. However, in comparison with another meta-analysis on the same topic, we not only compared remission rates after surgery and dopamine agonist withdrawal, but also biochemical control during medical therapy with postsurgical remission rates, also taking into account side effects and complications [18]. Although not extensively reported in the literature, multiple clinical and patient-reported outcomes were also assessed, including HRQoL and cost-effectiveness. Furthermore, a strength of this meta-analysis is that we included more studies (80 studies compared with 13 studies) in our analysis, partially because of a more extensive literature search, which increased the power and accuracy of the calculated estimates and made it possible to perform multiple sensitivity and subgroup analysis partially accounting for the heterogeneity in the included studies [18]. Although we assessed multiple outcomes, we could not assess the effect of both treatment modalities on all clinically relevant symptoms, such as gonadal status, and tumor shrinkage. Because not all surgical studies described evaluation of all pituitary axis after surgery, the reported results on postoperative hypopituitarism might be an underestimation. In addition, we performed sensitivity analysis including studies reporting remission rates after at least 1 year of follow-up . We did not perform analysis on longer (eg, 3, 5 years) follow-up results because of a lack of data in the included studies. Future studies should assess remission rates at longer follow-up because rates might change differently for both treatment modalities over time. Assessment of fertility and pregnancy as outcomes was beyond the scope of this systematic review and meta-analysis; we believe that this should be assessed in great detail in a separate study because of the complexity of treatment and timing of treatment before and during pregnancy. In addition, a high percentage of articles describe these outcomes in patients with hyperprolactinemia without an evident prolactinoma, which was an exclusion criterion for our systematic review and meta-analysis (7).

### Implementation of findings and future research

In contrast to the current guidelines, this meta-analysis of available literature indicates that surgery is indeed a viable alternative, first-line treatment for prolactinoma patients, especially for microprolactinoma patients, and warrants the revival of transsphenoidal surgery in the treatment algorithm and international guidelines for these patients. However, implementation of these results in clinical practice will be challenging. Because the majority of patients achieve normalized prolactin levels during treatment with dopamine agonists, there may be a preference for less-invasive medical treatment. However, treatment decisions should be made together with the patient and should not be based only on the high prolactin normalization levels during medical treatment, but also on side effects experienced and anticipated long-term need of dopamine agonists versus high disease remission rates after surgery, complications after surgery, and the effect of both treatment modalities on patient’s HRQoL and cost-effectiveness. Results on the latter are suggested by modeling studies and should be further explored in real-world cohorts and in future international randomized trials comparing both treatment modalities on cost-effectiveness. Because costs will largely differ per country, conclusions might be variable by country.

## Abbreviations

CI: confidence interval
HRQoL: health-related quality of life

## Additional Information

**Disclosure Summary:** The authors have nothing to disclose.

***Data Availability:*** All data generated or analyzed during this study are included in this published article or in the data repositories listed in References.
